# Changes of the retinal and choroidal vasculature in cerebral small vessel disease

**DOI:** 10.1038/s41598-022-07638-x

**Published:** 2022-03-07

**Authors:** Clara F. Geerling, Jan H. Terheyden, S. Magdalena Langner, Christine Kindler, Vera C. Keil, Christopher A. Turski, Gabrielle N. Turski, Maximillian W. M. Wintergerst, Gabor C. Petzold, Robert P. Finger

**Affiliations:** 1 Department of Ophthalmology, University Hospital Bonn, Ernst-Abbe-Str. 2, 53127 Bonn, Germany; 2 Department of Neurology, University Hospital Bonn, 53127 Bonn, Germany; 3 German Centre for Neurodegenerative Diseases (DZNE), 53127 Bonn, Germany; 4 Department of Radiology, Amsterdam UMC, Location VUmc, De Boelelaan 1117, 1081 HV Amsterdam, The Netherlands; 5 Division of Vascular Neurology, University Hospital Bonn, 53127 Bonn, Germany

**Keywords:** Diagnostic markers, Diagnostic markers

## Abstract

Cerebral small vessel disease (CSVD) is associated with changes in the retinal vasculature which can be assessed non-invasively with much higher resolution than the cerebral vasculature. To detect changes at a microvascular level, we used optical coherence tomography angiography which resolves retinal and choroidal vasculature. Participants with CSVD and controls were included. White matter lesions were determined on magnetic resonance imaging (MRI). The retinal and choroidal vasculature were quantified using swept-source optical coherence tomography angiography. Data were analysed using linear regression. We included 30 participants (18 females; patients, *n* = 20; controls, *n* = 10) with a mean age of 61 ± 10 years. Patients had a higher mean white matter lesion index and number of lesions than controls (*p* ≤ 0.002). The intraindividual deviation of choriocapillaris reflectivity differed significantly between age-matched patients (0.234 ± 0.012) and controls (0.247 ± 0.011; *p* = 0.029). Skeleton density of the deep retinal capillaries was significantly associated with the number of lesions on MRI (β = − 5.3 × 10^8^, 95%-confidence interval [− 10.3 × 10^8^; − 0.2 × 10^8^]) when controlling for age. The choroidal microvasculature and the deep retinal vascular plexus, as quantified by optical coherence tomography angiography, are significantly altered in CSVD. The value of these findings in diagnosing or monitoring CSVD need to be assessed in future studies.

## Introduction

Cerebral small vessel disease (CSVD) is one of the most frequent neurological disorders and is a major risk factor for the development of cognitive impairment and stroke^1,2^. With a prevalence of over 90% in elderly people and an expected rise due to population aging, complications of CSVD have enormous implications for society^3^. CSVD encompasses different pathologies that affect small cerebral vessels, but vascular alterations in kidneys, heart, the musculoskeletal system and the retina have also been reported^1^.

Retinal and cerebral vasculature have a similar embryological origin, anatomy and metabolism later in life^4^. These associations are reflected in an altered retinal vasculature in many neurodegenerative diseases^5^. Previous studies have shown retinal vascular changes in individuals with CSVD, including arteriolar narrowing and sclerosis as well as venular dilation and a reduction of overall vascular complexity using colour fundus photography^6,7^. Colour fundus photography is less sensitive to microvascular changes than optical coherence tomography angiography (OCT-A) which allows for perfusion assessment on a capillary level and additional assessment of the choriocapillaris, the innermost choroidal layer. Most recently, using OCT-A, reduced capillary density was reported in a cognitively impaired cohort of patients with CSVD^8^. Yet, the onset of these changes during the course of the disease as well as its exact anatomic localization in the retina and underlying choroid remain unclear. We thus investigated retinal and choroidal vascular changes using OCT-A in patients with CSVD and controls.

## Materials and methods

### Study participants

Study participants were identified at the Department of Neurology at the University Hospital Bonn and considered eligible if they had a recent magnetic resonance imaging (MRI) (≤ 6 months) confirming either CSVD (cases) or absence of any cerebral pathology (controls). Potential participants were contacted a maximum of three times by phone and informed of the study. The study was approved by the human research ethics committee of the University Hospital Bonn, North Rhine Westphalia, Germany (consecutive number 281/17). The study adhered to the tenets of the Declaration of Helsinki. Written informed consent was obtained from every participant. Data were collected from January 2017 to May 2019.

### Clinical assessment

All subjects underwent an ophthalmic assessment and ocular imaging by two trained medical examiners at the Department of Ophthalmology at the University Hospital Bonn. Medical history was obtained using a standardized questionnaire. Cognitive assessment was performed with the Montreal Cognitive Assessment test (MoCA), a tool to measure global cognitive function and screen for various types of dementia^9^. All measurements were acquired at the same day as the ophthalmic assessment. Exclusion criteria were any eye diseases interfering with the ophthalmic assessments or structural neurological anomalies.

### Ocular image acquisition and analysis

OCT-Angiography methology and results are reported in line with the APOSTEL recommendations^10^. Fundus images were acquired in both eyes without pupil dilatation with one single swept-source OCT-A device at 100 000 A- scans/second at 1060 nm wavelength (Zeiss PLEX Elite 9000; Carl Zeiss Meditec, Dublin, California, USA). Optical coherence tomography is a non-invasive, noncontact imaging method based on local interference between an object's signal and a reference signal^11^. It has recently been extended to OCT-Angiography, which allows for the non-invasive visualization of the retinal vasculature by detecting blood flow based on the variability in OCT amplitude and phase signal over time^12^.

**Figure 1:**
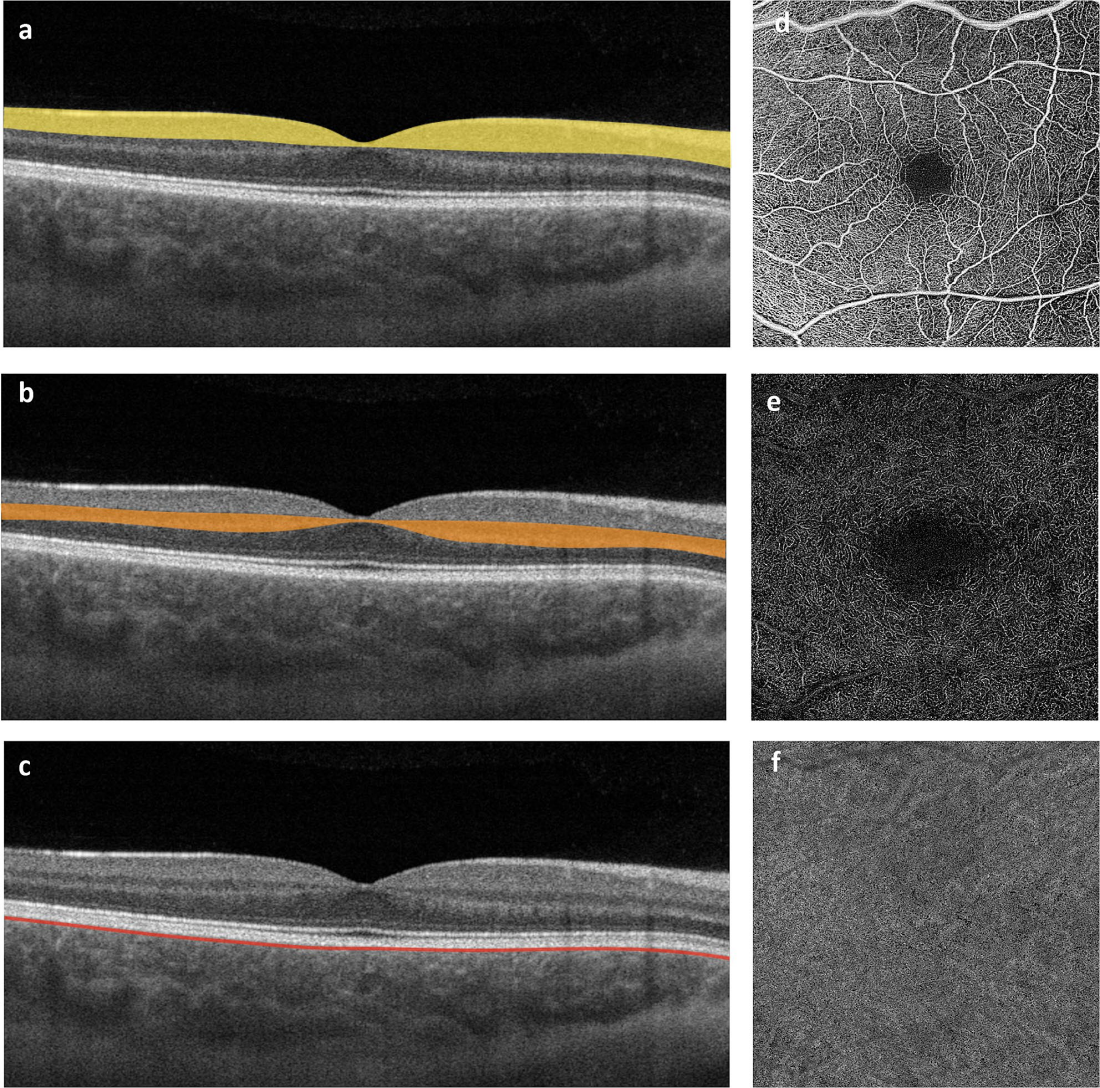
Anatomic localisation and segmentation boundaries of the vascular plexus investigated on macular OCT B-scans (**a**, superficial retinal layer; **b**, deep retinal layer; **c**, choriocapillaris) and respective OCT-A en face images (coronal plane; **d**, **e**, **f**).

Scans with a pattern size of 6 × 6 mm of the macula were performed. Every A-line was performed by 500 A-scans per B-scan and two repetitions on 500 B-scans. It was acquired over a depth of 3 mm and contained 1024 × 1024 pixel (6 µm/pixel). Images with insufficient quality (signal strength index < 8) were excluded from the analysis based on the evaluation of two readers (CFG, JHT). OCT-A images were generated using an optical microangiography complex algorithm and decorrelation tail artifacts removed by a general sliding slab method^13^. OCT-A en face images of the superficial retinal vascular layer (spanning the nerve fibre, ganglion cell, and inner plexiform layers) and deep retinal layer (spanning the inner nuclear and outer plexiform plus Henle fibre layer) and the choriocapillaris (10–40 µm below retinal pigment epithelium-fit segmentation) layer were created using the maximum projection of each particular slab within the artefact-corrected volume. The superficial and deep retinal layer were defined according to standard OCT-A nomenclature (Fig. 1)^14^. Layer segmentation was performed automatically, using the proprietary algorithm of the device.

For quantitative image analysis of the 6 × 6 mm scans of the macula, images were processed with Fiji^15^ (an expanded version of ImageJ, version 1.52)^16^. For image binarization, the automated thresholding algorithms according to Li (superficial retinal plexus) and Otsu (deep retinal plexus) were used^17,18^. Vessel density, skeleton density and vessel diameter index of the superficial and deep retinal vessel layers were calculated based on the binarized and skeletonized images. Vessel density and skeleton density are relative values describing the total length of the vessels. Skeletonized vessels have a thickness of 1 pixel, reducing the impact of larger-diameter vessels (arterioles and venules) on the overall density and increasing the impact of capillary changes on the parameter. Vessel diameter index describes the total area occupied by the vessels^13^. Choriocapillaris perfusion changes were quantified using mean signal intensity (i.e. reflectivity) and its standard deviation^13^. In addition, the choriocapillaris en face images were binarized using the Phansalkar method and the number, average size and distribution of flow voids were compared^19,20^.

### Brain magnetic resonance imaging and analysis

For brain imaging analysis, we used axial whole-brain fluid-attenuated inversion recovery (FLAIR) images from our radiological database (Supplementary Table 1). All patients had received 1.5 or 3 T MRI with an 8-channel head coil (Philips Ingenia 1.5 T and Achieva TX 3 T; Philips Healthcare, Best/The Netherlands) for a clinical indication between January, 2017 and May, 2019. The FLAIR images were used for microvascular leukoencephalopathy assessment according to Fazekas et al*.*^21^ by two board-certified neuroradiologists blinded to the clinical condition of the patient.

Burden of white matter lesions was estimated automatically using the “lesion prediction algorithm” as implemented in the Lesion Segmentation Toolbox version 2.0.15 for SPM12^22^. This algorithm is based on a binary classifier in the form of a logistic regression model based on the data of 53 multiple sclerosis patients. It uses a similar lesion belief map as the “lesion growth algorithm” and a spatial covariate that takes into account voxel specific changes in lesion probability^23^. New images are segmented using the parameters of this model adaptation, which provides one voxel each for the estimation of the lesion probability. The algorithm is considered to be a valid and robust method for the detection of white matter lesions of diverse origins^24^.

The lesion prediction algorithm requires only FLAIR images. Therefore, the full brain 2D FLAIR volumes of the participants were applied to segment the individual white matter hyperintensities. The segmentation accuracy of the white matter hyperintensity volumes and numbers was checked by visual inspection. Additionally, lesion filling for all 2D FLAIR volumes was performed using the same toolbox. The individually filled in 2D FLAIR volumes were then segmented into the tissue classes white matter, grey matter and cerebrospinal fluid using the standard segmentation algorithm provided by SPM12. The total intracranial volume was calculated as the sum of the three tissue classes. To obtain the white matter hyperintensity volume as a percentage of the intracranial volume (WMI), the native lesion volumes were divided by total intracranial volume and multiplied with 100.

### Statistical analysis

Statistical analysis was performed using SPSS Statistics for Windows, version 26 (IBM, Armonk, New York, USA) and R version 4.0.5 (R Core Team, Vienna, Austria). In participants with two eligible study eyes, the mean values of the respective OCT-A parameters were calculated per participant. WMI values were logarithmically transformed since the original parameter was not normally distributed. CSVD and control participants were compared using the Mann–Whitney-U test on the basis of non-normal distributions. The ability of OCT-A parameters to discriminate between participants with CSVD (Fazekas score ≥ 1) and without CSVD were assessed using receiver operating characteristic (ROC) curve analyses in the overall cohort. Associations of MRI parameters with OCT-A parameters were examined using multiple linear regression analysis in the complete sample and in subgroup analyses of age-matched cases and controls as well as cases only. P-values < 0.05 were considered statistically significant.

## Results

**Figure 2:**
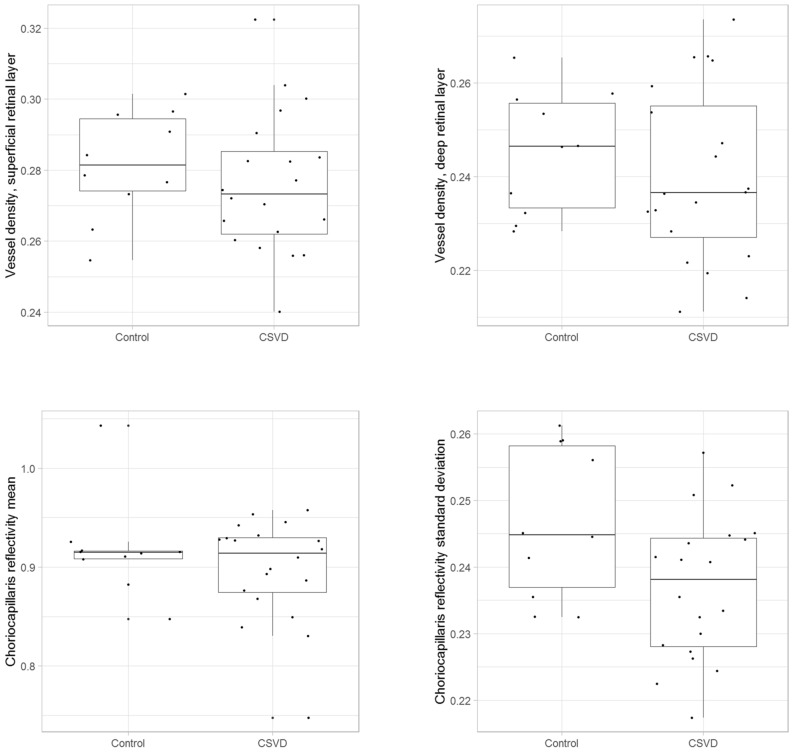
Boxplots showing the distributions of OCT-A parameters which represent the vessel plexus investigated in participants with CSVD and controls.

62 Participants were recruited for clinical assessment. We excluded 32 subjects due to ocular disease (n = 18), poor OCT-A image quality (n = 9), and neurologic abnormalities (n = 5). Thus, 30 participants (18 women and 12 men) with an average age of 61 ± 10 years (range: 47 to 78 years) were included (Table 1). Ten participants were healthy controls (Fazekas score 0) and 20 participants had CSVD (Fazekas score 1, *n* = 11; Fazekas score ≥ 2, *n* = 9). The average MoCA score was 25 ± 3 points, with no significant difference between cases and controls (*p* = 0.948). In the total sample, choriocapillaris reflectivity standard deviation differed significantly between cases and controls (*p* = 0.039; Fig. 2).

**Table 1:** Demographic MRI and OCT-A parameters for the overall sample and by CSVD cases and controls.

	All	Controls	Age-matched cases	*P* value^1^	All cases	*P* value^2^
Sex (f;m)	18; 12	6; 4	5; 5		12; 8	
Age	61 ± 10	58 ± 5	60 ± 10	0.853	63 ± 11	0.475
Fazekas score	0; 1; 2; 3	0	1; 2; 3		1; 2; 3	
MoCA	25 ± 3	25 ± 3	26 ± 3	0.529	25 ± 4	0.948
Vessel Density, superficial	0.278 ± 0.018	0.281 ± 0.015	0.279 ± 0.019	0.579	0.276 ± 0.020	0.373
Vessel Diameter Index, superficial	1,956,055 ± 68,017	1,979,802 ± 30,639	1,949,547 ± 64,953	0.075	1,944,182 ± 78,558	0.100
Vessel Density, deep	0.242 ± 0.017	0.245 ± 0.013	0.239 ± 0.021	0.529	0.240 ± 0.019	0.530
Vessel Diameter Index, deep	1,553,981 ± 64,138	1,571,945 ± 59,464	1,543,678 ± 76,799	0.436	1,544,999 ± 65,947	0.328
Choriocapillaris reflectivity, mean	0.905 ± 0.051	0.918 ± 0.050	0.898 ± 0.043	0.631	0.899 ± 0.052	1.000
Choriocapillaris reflectivity, SD	0.240 ± 0.01179	0.247 ± 0.011	0.234 ± 0.012	*0.029	0.237 ± 0.011	*0.039
NOL	12 ± 8	6 ± 6	16 ± 8	*0.002	16 ± 8	*0.001
WMI	27.34 × 10^–4^ ± 44.61 × 10^–4^	2.58 × 10^–4^ ± 2.57 × 10^–4^	44.08 × 10^–4^ ± 53.31 × 10^–4^	* < 0.0001	39.71 × 10^–4^ ± 50.50 × 10^–4^	*0.0001

MoCA, Montreal Cognitive Assessment; NOL, number of white matter hyperintensity lesions on magnet resonance imaging; WMI, White Matter Lesion Index; SD, standard deviation; ^1^Comparison of age matched cases to controls, ^2^Comparison of all cases to controls.

The numbers are marked with asterisks to highlight statistical significance.

The number and average size of flow voids did not differ between CSVD cases and controls (*p* = 0.681 and 0.307, respectively) but the distribution of noticeably high or low numbers of flow voids was significantly different between both groups (*p* = 0.040) and individuals with CSVD had on average either more or fewer choriocapillaris flow deficits than control participants. In addition, an analysis of the logarithmic association between the size of flow deficits and their number of occurrence using linear regression as suggested by Spaide revealed a significant difference in the intercepts (*p* = 0.005)^20^. The unadjusted area under the ROC curve to detect participants with CSVD was significant for the choriocapillaris reflectivity standard deviation on OCT-A (0.735, 95%-confidence interval [0.544; 0.926], *p* = 0.039) (Supplementary Table 2 Supplementary Fig. 1). The logarithmically transformed WMI values as well as number of lesions were significantly associated with vessel density, vessel diameter index in both superficial and deep plexus as well as choriocapillaris parameters in unadjusted analyses (Supplementary Fig. 2). None of these associations remained significant when controlling for age (Table 2).

**Table 2:** Associations of MRI and OCT-A parameters for the overall sample in multiple linear regression analysis.

MRI parameter	OCT-A parameter	Β	95% CI	*P* value
WMI	Vessel density, superficial	− 17.9	[− 33.3; − 2.5]	*0.024
	Vessel diameter index, superficial	− 5.3 × 10^–6^	[− 9.0 × 10^–6^; − 1.0 × 10^–6^]	*0.012
NOL	Vessel density, superficial	− 231.9	[− 386.1; − 77.7]	*0.005
	Skeleton density, superficial	− 6.6 × 10^8^	[− 1.2 × 10^9^; − 1.5 × 10^8^]	*0.013
	Vessel diameter index, superficial	− 5.6 × 10^–5^	[− 9.8 × 10^–5^; − 1.3 × 10^–5^]	*0.013
	Vessel density, deep	− 183.9	[− 363.4; − 4.4]	*0.045
	Choriocapillaris reflectivity, mean	− 63.3	[− 122.3; − 4.3]	*0.036
**Controlled for age**				
WMI	Vessel density, superficial	− 7.7	[− 24.6; 9.2]	0.358
	Vessel diameter index, superficial	− 3.1 × 10^–6^	[− 7.0 × 10^–6^;9.8 × 10^–7^]	0.128
NOL	Vessel density, superficial	− 157.9	[− 334.8; 19.0]	0.078
	Skeleton density, superficial	− 4.0 × 10^8^	[− 9.7 × 10^8^; 1.7 × 10^8^]	0.158
	Vessel diameter index, superficial	− 3.6 × 10^–5^	[− 8.2 × 10^–5^; 9.0 × 10^–6^]	0.112
	Vessel density, deep	− 147.1	[− 312.0; 17.8]	0.078
	Choriocapillaris reflectivity, mean	− 49.5	[− 104.2; 5.3]	0.075

NOL, number of white matter hyperintensity lesions on magnet resonance imaging; SD, standard deviation; WMI, White Matter Lesion Index.

The numbers are marked with asterisks to highlight statistical significance.

Subsequently, two subgroup analyses were performed. Firstly, in an age-matched analysis of 10 cases and 10 controls, choriocapillaris reflectivity standard deviation differed significantly between the groups (Table 1). Similarly to the overall cohort, there were no significant differences regarding number and size of flow voids (*p* = 0.481 and 0.190, respectively) but the above mentioned, significant difference in the distribution of flow voids remained present (*p* = 0.049) and the difference in the linear regression intercepts remained significant (*p* = 0.029). Secondly, in a subgroup of patients only (*n* = 20), number of lesions was significantly associated with five OCT-A parameters of the superficial and deep retinal plexus (Table 3). After controlling for age, skeleton density of the deep retinal layer remained significant (*p* = 0.041). WMI was not significantly associated with any of the OCT-A parameters in the CSVD subgroup.

**Table 3:** Association of NOL on MRI and OCT−A parameters for the subgroup analysis of cases in multiple linear logistic regression analysis.

Parameter	Β	95% CI	*P* value
Vessel density, superficial	− 206.0	[− 372.5; − 39.6]	*0.018
Skeleton Density, superficial	− 8.0 × 10^8^	[− 13.8 × 10^8^; − 2.2 × 10^8^]	*0.010
Vessel diameter index, superficial	− 3.9 × 10^–5^	[− 8.4 × 10^–5^; 0.6 × 10^–5^]	0.086
Vessel Density, deep	− 202.5	[− 384.5; − 20.6]	*0.031
Skeleton Density, deep	− 6.1 × 10^8^	[− 11.9 × 10^8^; − 4.0 × 10^8^]	*0.037
Vessel Diameter Index, deep	− 5.3 × 10^–5^	[− 10.5 × 10^–5^; − 0.1 × 10^–5^]	*0.045
Choriocapillaris reflectivity, mean	− 64.5	[− 132.0; 3.0]	0.06
Choriocapillaris reflectivity, SD	72.1	[− 281.4; 425.7]	0.673
**Controlled for age**			
Vessel Density, superficial	− 124.0	[− 317.8; 69.9]	0.195
Skeleton Density, superficial	− 5.2 × 10^8^	[− 12.5 × 10^8^; 2.1 × 10^8^]	0.152
Vessel Density, deep	− 157.2	[− 323.7; 9.2]	0.062
Skeleton Density, deep	− 5.3 × 10^8^	[− 10.3 × 10^8^; − 0.2 × 10^8^]	*0.041
Vessel Diameter Index, deep	− 3.8 × 10^–5^	[− 8.7 × 10^–5^; 1.2 × 10^–5^]	0.126

NOL, number of white matter hyperintensity lesions on magnet resonance imaging; SD, standard deviation.

The numbers are marked with asterisks to highlight statistical significance.

## Discussion

In this study we found choroidal perfusion altered in CSVD compared to healthy controls which has not been described in CSVD previously. The extent of CSVD on MRI was associated with a reduced blood flow in the capillaries of the deep retinal plexus independent of age-related changes. Our results indicate that the high-resolution assessment of the choroidal and retinal microvasculature using OCT-A might aid in detecting and monitoring CSVD but additional studies are needed to better characterize these associations.

Lee et al*.*^8^ published a study on retinal microvascular changes in patients with cognitive impairment due to CSVD (25 cases and 15 controls). In their study, capillary density in the radial peripapillary retinal plexus was significantly reduced compared to controls and the peripapillary capillary density on OCT-A was associated with a MRI-based CSVD disease score. The results are in line with the age-independent, significant reduction of capillary flow in the deep retinal plexus in our cohort. However, Lee et al*.*^8^ did not consider any choroidal parameters, nor did they investigate regions other than the peripapillary area or features of specific anatomic layers. Distinguishing alterations in distinct anatomical layers seems particularly important because in our cohort, only blood flow in the deep retinal plexus was significantly associated with MRI lesion count independently of age. Wang et al*.*^25^ recently reported a vessel density reduction in the superficial retinal capillary plexus in CSVD patients but did not consider any choroidal parameters either. Abdelhak et al.^26^ used structural OCT data to assess the retinal arterioles in individuals with stroke secondary to sporadic CSVD. They identified an increase in ratio between wall and lumen ratio in patients with CSVD, supporting that the retinal vasculature is altered in CSVD. Our study supports this assumption and complements it with the more detailed view of the retinal capillaries and the choriocapillaris visualized by OCT-A. Lee et al.^27^ investigated structural retinal changes in 15 individuals with ischemic stroke using OCT but did not identify any significant structural alterations that may result from the capillary flow impairment identified in our study. Overall, the reported anatomic locations of retinal vessel flow pathologies in CSVD remain inconsistent but we have newly identified alterations in the superficial proportion of the choroidal layer in these patients.

In agreement with our results investigating sporadic CSVD, patients with cerebral autosomal dominant arteriopathy with subcortical infarcts and leukoencephalopathy, a hereditary subtype of CSVD, were found to have dilated arteriolar and venous diameters as well as a reduced vessel density in the deep retinal plexus on OCT-A^28^.

Most previous research on ocular manifestations of CSVD was based on fundus photography. Since this method is limited to a resolution of arterioles and venules, alterations of the capillary system or the choroid cannot be assessed^29^. In the population-based Rotterdam study, Ikram et al*.*^6^ examined 490 participants by means of funduscopy. Larger venule diameters were related with a marked progression of periventricular and subcortical white matter lesions and incident lacunar infarcts on MRI over three to five years. Similarly, Mutlu et al*.*^30^ and Qui et al*.*^31^ observed arteriolar narrowing and venular dilatation to be pronounced with larger white matter hyperintensities and lacunar infarcts. More recently, McGrory et al*.*^7^ described a significant correlation between white matter hyperintensities and a reduction in arteriolar fractal dimension in the Lothian Birth Cohort and the Mild Stroke Study. Our results confirm the presence of retinal changes in CSVD. Using modern, non-invasive ophthalmic imaging technology, we were able to assess spatially-resolved changes within the retinal and the choroidal microvasculature. Assuming that the smallest vessels are those with the earliest changes, we would conject that any vascular pathology present at the time of retinal imaging has been detected.

The strengths of our study include the thorough screening for concurrent neurological or ocular comorbidities, the use of high-resolution MRI, a complete ophthalmic assessment, the use of state-of-the-art high-resolution swept source OCT-A imaging and the use of standardized and published automated image analysis for both MRI and OCT-A. The limitations of our study include foremost its relatively small sample size. As our study was designed as an exploratory study, confirmatory studies with larger sample sizes are needed. The significant age difference between the cohorts, limits the interpretation of discrimination power within our sample. Also, we included only the mean values of OCT-A parameters in participants with two eligible eyes in the analysis. The quantification of choriocapillaris information is highly dependent on the OCT instrument, scan protocol, and signal processing^32–35^, which may limit comparability to other studies and the applicability of our results to other settings. The technology used may limit the inter-individual comparability of choriocapillaris reflectivity values but our analysis of choriocapillaris flow voids was in line with the findings based on reflectivities and supports our overall interpretation of findings. Different MRI devices and sequences were used in a clinical routine setting which could impact the comparability of individual volumes. In order to ensure comparability we used the lesion segmentation tool and “lesion prediction algorithm” segmentation method, which has proven good performances within and across scanners for white matter hyperintensity segmentation in a multicentre dataset^36^. Non-neurological and non-ocular comorbidities were not assessed which might have affected our findings. The time lag between MRI- and OCT-imaging might have led to an underestimation of effect size as cerebral CSVD lesions might have progressed. Lastly, we did not correct for multiple testing as this was an exploratory study, which might lead to spurious associations. Thus, any findings need confirmation in additional studies.

In conclusion, we found significant alterations of the choroidal and the retinal perfusion in CSVD which were independent of age. Differences between participants with CSVD and healthy controls were pronounced in the choroid while the extent of CSVD was most highly associated with alterations in the deep retinal vascular plexus. These finding need confirmation in future trials and might ultimately help in earlier detection and better monitoring of CSVD.

## Supplementary Information

Supplementary Information.

## Data Availability

The data proving the main findings of the study are contained within the manuscript. Additional data are available upon reasonable request from the University Hospital Bonn, Department of Ophthalmology at phone number +4922828715505.
