# Supplementary material for: Changes of the retinal and choroidal vasculature in cerebral small vessel disease
Source: Sci Rep. 2022 Mar 7;12:3660. doi: 10.1038/s41598-022-07638-x (PMC8901619; doi:10.1038/s41598-022-07638-x)

Changes of the retinal and choroidal vasculature in cerebral small vessel disease

- Supplement -

Clara F. Geerling†, Jan H. Terheyden†, S. Magdalena Langner, Christine Kindler, Vera C. Keil, Christopher A. Turski, Gabrielle N. Turski, Maximillian W.M. Wintergerst, Gabor C. Petzold*, Robert P. Finger*

Supplementary Table 1. Imaging parameters

MRI	Sequence	Orientation	Slice thickness	Matrix	TR	TE	IT
3 Tesla	2D FLAIR	axial	6mm; no gap	512×512	12 s	140 ms	2850 ms
1.5 Tesla	2D FLAIR	axial	6mm; no gap	352×352	11 s	140 ms	2800 ms

Abbreviations: TE, echo time; IT, inversion time; TR, repetition time

Supplementary Table 2. Results of receiver operating curve-analysis in the overall cohort

Parameter	AUC	95%-Confidence interval	P-value
WMI	0.920	[0.820;1.0]	*< 0.0001
NOL	0.858	[0.713;1.0]	*0.002
Choriocapillaris reflectivity, SD	0.735	[0.544;0.926]	*0.039

Abbreviations: CI, confidence interval; AUC, area under the receiver operating curve; NOL, number of white matter hyperintensity lesions on magnet resonance imaging; SD, Standard deviation

Supplementary Figure 1. Receiver operating characteristic curve for choriocapillaris reflectivity standard deviation, white matter lesion index and number of lesions

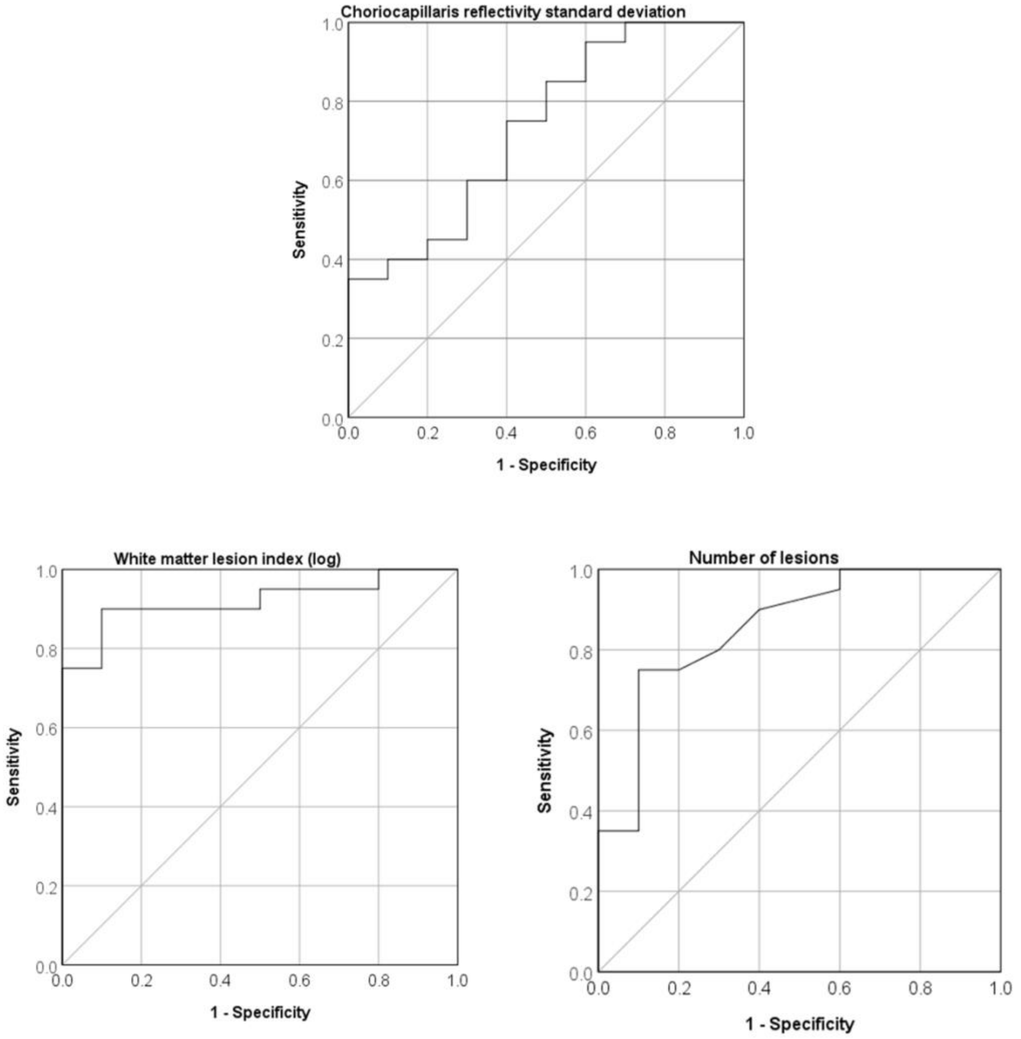

Supplementary Figure 2. Graphical representation of associations between the investigated OCT-A and MRI parameters. Triangles refer to participants with CSVD and circles to control participants

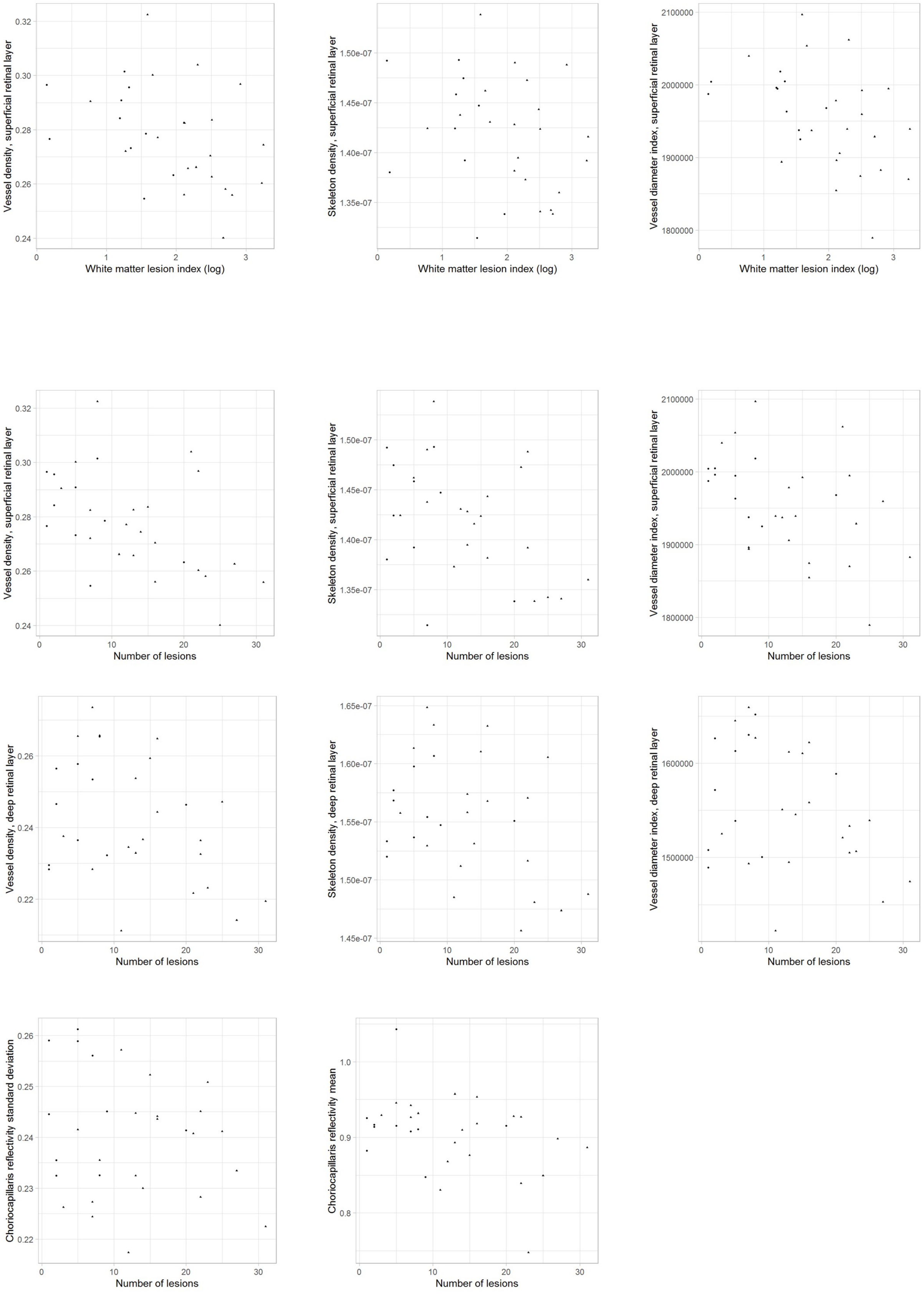

Supplement: Supplementary file 1 — Supplementary Information. [file 41598_2022_7638_MOESM1_ESM.pdf]
